# T Cells Engaging the Conserved MHC Class Ib Molecule Qa-1^b^ with TAP-Independent Peptides Are Semi-Invariant Lymphocytes

**DOI:** 10.3389/fimmu.2018.00060

**Published:** 2018-01-25

**Authors:** Elien M. Doorduijn, Marjolein Sluijter, Bianca J. Querido, Ursula J. E. Seidel, Claudia C. Oliveira, Sjoerd H. van der Burg, Thorbald van Hall

**Affiliations:** ^1^Department of Medical Oncology, Leiden University Medical Center (LUMC), Leiden, Netherlands

**Keywords:** CD8^+^ T cells, Qa-1^b^, non-classical MHC, peptide transporter TAP, T cell receptor, thymus

## Abstract

The HLA-E homolog in the mouse (Qa-1^b^) is a conserved MHC class Ib molecule presenting monomorphic peptides to germline-encoded natural killer receptor CD94/NKG2A. Previously, we demonstrated the replacement of this canonical peptide by a diverse peptidome upon deficiency of the TAP peptide transporter. Analysis of this Qa-1^b^-restricted T cell repertoire against these non-mutated neoantigens revealed characteristics of conventional hypervariable CD8^+^ T cells, but also of invariant T cell receptor (TCR)αβ T cells. A shared TCR Vα chain was used by this subset in combination with a variety of Vβ chains. The TCRs target peptide ligands that are conserved between mouse and man, like the identified peptide derived from the transcriptional cofactor Med15. The thymus selection was studied in a TCR-transgenic mouse and emerging naïve CD8^+^ T cells displayed a slightly activated phenotype, as witnessed by higher CD122 and Ly6C expression. Moreover, the Qa-1^b^ protein was dispensable for thymus selection. Importantly, no self-reactivity was observed as reported for other MHC class Ib-restricted subsets. Naïve Qa-1^b^ restricted T cells expanded, contracted, and formed memory cells *in vivo* upon peptide vaccination in a similar manner as conventional CD8^+^ T cells. Based on these data, the Qa-1^b^ restricted T cell subset might be positioned closest to conventional CD8^+^ T cells of all MHC class Ib populations.

## Introduction

The non-classical mouse MHC class I (MHC-I) molecule Qa-1^b^ and its human homolog HLA-E are non-polymorphic MHC molecules with important functions in innate immunity. These proteins bind and present signal peptides of classical MHC-I molecules at the cell surface and, as such, act as indirect sensors for the normal expression of MHC-I molecules (1, 2). This signal peptide dominantly accommodated in the groove of Qa-1^b^ is called Qdm, for Qa-1 determinant modifier (3, 4), and its amino acid sequence AMAPRTLLL is amazingly conserved among mammalian species (5). The Qdm/Qa-1^b^ complex serves as a ligand for the germ-line encoded heterodimeric CD94/NKG2A receptors expressed on NK cells and activated CD8^+^ T cells and transduces inhibitory signals to these lymphocytes (5–7). This innate role of Qa-1^b^ and HLA-E has been well characterized.

Although the self-peptide Qdm is the dominant monomorphic peptide presented by Qa-1^b^ and HLA-E, other microbial peptides are capable of stabilizing Qa-1^b^ and HLA-E. Examples of these are a heat shock protein 60 derived peptide (8), insulin peptides (9, 10), and a peptide from the *Salmonella enterica* bacterium (11). Such examples have also been described for the human HLA-E homolog, where peptides were found from an endogenous multidrug resistance transporter protein (12) and from the pathogens Cytomegalovirus, Hepatitisvirus, *S. enterica* bacterium, and *Mycobacterium tuberculosis* (13–17). For some of these alternate peptides, specific CD8^+^ T cells have been identified, showing that Qa-1^b^ and HLA-E are also involved in adaptive immunity to present antigens (11, 14, 16, 18–20). Of note, an interesting population of Qa-1^b^ regulatory CD8^+^ T cells have been described targeting self-peptides and dampening auto-immunity (21–23). The engagement by T cell receptor (TCR) is not surprising as both molecules fold like conventional MHC-I molecules and support interaction with CD8 (7, 24, 25).

Previously, we and others have reported on the presentation of endogenous peptides by Qa-1^b^ on cells with a defect in the antigen-processing machinery (26, 27). Defects in the antigen-processing machinery, as reported for the peptide transporter TAP, the peptide-editor tapasin, or the ER-resident amino peptidase ERAAP, results in failure to present Qdm and, consequently, allows the display of alternative peptides from endogenous sources. Viruses and tumor cells regularly downregulate these processing components and thereby evade immune surveillance by CD8^+^ T cells. Mass spectrometry analysis of peptides from TAP-deficient tumor cells revealed a large and diverse repertoire of alternative peptides (27). A similar diversity was found for HLA-E (28). Cells deficient for the aminopeptidase ERAAP presented the novel peptide FL9 in the context of Qa-1^b^ (26). These alternative peptides were immunogenic in that they induced CD8^+^ T cell responses, although the donor proteins were of self-origin.

Here, we studied common characteristics of Qa-1^b^-restricted T cells that recognize these alternative peptides on TAP-deficient target cells. We demonstrate that these T lymphocytes display features of semi-invariant T cells: 1. a conserved TCR Vα segment is used, whereas their CDR3 and the TCRβ chains were diverse; 2. the Qa-1^b^ presented peptide ligands were shared by mouse, human, and monkey cells; 3. the generation in the thymus was inefficient in a TCR-transgenic mouse, and 4. the thymus education was independent of Qa-1^b^. Importantly, the emerging T cell repertoire in the periphery still exhibited strong clonal expansion and effector functions after peptide vaccination. We furthermore show that Qdm-reactive T cells are strictly deleted from the repertoire. Based on our results, Qa-1^b^-restricted CD8^+^ T cells need to be positioned between conventional hypervariable TCRαβ T cells and real invariant T cells like NKT and MAIT cells.

## Materials and Methods

### Cell Lines and Mice

The human tumor cell lines HeLa and T2 and the monkey COS-7 cells were derived from ATCC. Gene transfer of *H-2T23* (Qa-1^b^) and the TAP-inhibitor *UL49.5* (BTIP) was performed by retroviral transduction as previously described, as well as the generation and culture of T cell clones (27). Dendritic cells were derived from bone marrow by culture for 1 week in the presence of IL-4 and GM-CSF (29). All cells were cultured in complete IMDM medium (Invitrogen, Carlsbad, CA, USA) containing 8% heat-inactivated FCS (Gibco), 100 U/ml penicillin, 100 µg/ml streptomycin and 2 mM l-glutamine (Invitrogen) at 37°C in humidified air with 5% CO_2_.

C57BL/6 mice were purchased from Charles River (L’Arbresle, France). The TAP1^−/−^ mice (The Jackson Laboratory stock no. 002944), Rag1^−/−^ mice (The Jackson Laboratory stock no. 003729) and the Qa-1^b−/−^ mice (The Jackson Laboratory stock no. 007907) were bred in our own facility. Rag1^−/−^ mice were a kind gift from Dr. Frank Staal (LUMC, Leiden) and Qa-1^b−/−^ mice were kindly provided by Marc Vocanson (Lyon, France). Mice were housed in individually ventilated cages and used at 6–12 weeks of age. All animal experiments were controlled by the animal welfare committee (IvD) of the Leiden University Medical Center and approved by the national central committee of animal experiments (CCD) under the permit number AVD116002015271.

### Generation of TCR-Transgenic Ln12 Mouse

The Ln12 TCR-transgenic mouse strain was generated by transgenesis of the TCRα and TCRβ genes of the Ln12 T cell clone. The TCRα and TCRβ chains were separately cloned into pCRII-TOPO plasmid vectors (Invitrogen) using RT-PCR and sequenced. Expression of TCRs was performed by retroviral transduction of the TCR genes in C57BL/6 splenocytes using pMX vectors (Ton Schumacher, NKI, Amsterdam). For transgenesis, the two chains were separately cloned into VA-hCD2 vectors (30) and inserts were subsequently injected in C57BL/6 oocytes. DNA from tails of founder mice was analyzed by PCR for presence of the constructs, using the forward primer in the CD2 cassette 5′-GGT GTG GAC TCC ACC AGT CTC ACT TC-3′ and the reverse primer in the TCRα 5′-TAA CTG GTA CAC AGC AGG-3′ or TCRβ region 5′-CAA ACA AGG AGA GAC CTT G-3′ (Invitrogen). One transgenic founder strain (no 30) was selected for further breeding, since this mouse displayed highest TCR-Vβ10 protein expression on T cells as determined by specific antibody (clone B21.5, Biolegend). All experiments were performed with ln12 transgenic mice backcrossed to Rag1^−/−^ mice and, for some experiments, subsequently to Qa-1^b−/−^ mice.

### Peptide Vaccination, *In Vivo* Cytotoxicity Assay, and Adoptive T Cell Transfer

Peptide vaccination was performed at day 1 and day 8 after T cell transfer with 50 µg of the Med15 or Qdm peptide together with the imiquimod-containing cream Aldara, as described before (31). For the *in vivo* cytotoxicity assay, immunized mice were challenged with three differentially CFSE-labeled populations of splenocytes, unloaded or loaded with the Med15 or Qdm peptide, as described before (31). Two days after transfer of target cells, mice were sacrificed and spleens were analyzed for the presence of target cells. For T cell transfer assays, single cell suspensions were made by mechanical disruption of spleen and lymph nodes from ln12-transgenic mice. Cells were passed through nylon wool to enrich for T cells and 1 × 10^6^ CD8^+^ T cells were injected in 200 µl PBS intravenously in recipient mice.

### Flow Cytometry Analysis

For flow cytometry analysis, single cell suspensions were stained in 0.1% BSA/PBS with antibodies against CD4 (clone RM4-5), CD8 (53.6-7), CD3 (145-2C11), TCRβ (H57-597), Vβ10 (B21.5), CD25 (PC61), CD62L (MEL-14), CD122 (TM-b1), Ly6C (HK1.4), CD5 (53-7,3), and CD44 (IM7) which were purchased from eBioSciences, Biolegend, or Becton and Dickinson (BD). For Qa-1^b^ staining, cells were first incubated with Qa-1^b^-biotin (6A8.6F10.1A6) and subsequently with streptavidin-APC. TCR Vβ usage was determined by flow cytometry using the Mouse Vβ TCR screening panel (BD Pharmingen). Intracellular cytokine staining was performed using the ICS kit from BioLegend according to manufactures protocol. In short, cells were permeabilized for 20 min with the fixation buffer on ice, washed twice in washing buffer, and thereafter stained for IFNγ (XMG1.2, Biolegend). Cells were analyzed on a FACS Calibur or Fortessa (BD), and all analysis was performed using FlowJo software (Treestar).

### *In Vitro* T Cell Stimulations

For *in vitro* recognition of T cells from the original T cell clone, 5,000 T cells were co-cultured overnight with titrating amounts of tumor cells in 96-well *U*-bottom plates. For peptide stimulation, 1 µg/ml peptide was added to the wells and at day 1 and day 2. These peptides were tested: Med15 (RLIIHFRDI), Qdm (AMAPRTLLL), FAP (FAPLPRLPTL), HSP60 (GMKFDRGYI), and FL9 (FYAEATPML). Splenocytes transduced with cloned TCR genes from Ln12 T cell clone or control retrovirus were used as effector cells (100,000 per well) and stimulated with 1 µg/ml peptide in 96-well *U*-bottom plates. Overnight IFNγ production by T cells was determined by ELISA. T cell stimulation assays with freshly isolated transgenic Ln12.Rag1^−/−^ T cells were performed with pooled spleen and lymph node cells. CD8^+^ T cells were purified using the CD8^+^ enrichment kit (BD) and labeled with 5 µM CFSE. A total of 100,000 cells were treated in a 96-well with various stimuli in the presence of 10 CU IL-2/ml. For αCD3/αCD28 stimulation, wells were coated overnight with 0.5 µg/ml αCD3 (145-2C11, BD). The next day, coated wells were washed with medium and cells were added with 2 µg/ml αCD28 (37.51, Biolegend). Cells were analyzed by flow cytometry and supernatants were analyzed for cytokine release measured by IFNγ ELISA.

### Statistics

Statistical analyses were performed in GraphPad Prism, version 7.0. *P*-values <0.05 were considered statistically significant and indicated with *, *p* < 0.01 with **, and *p* < 0.001 with ***.

## Results

### Conserved TCR Vα Usage by Qa-1^b^-Restricted CD8^+^ T Cells

**Figure 1:**
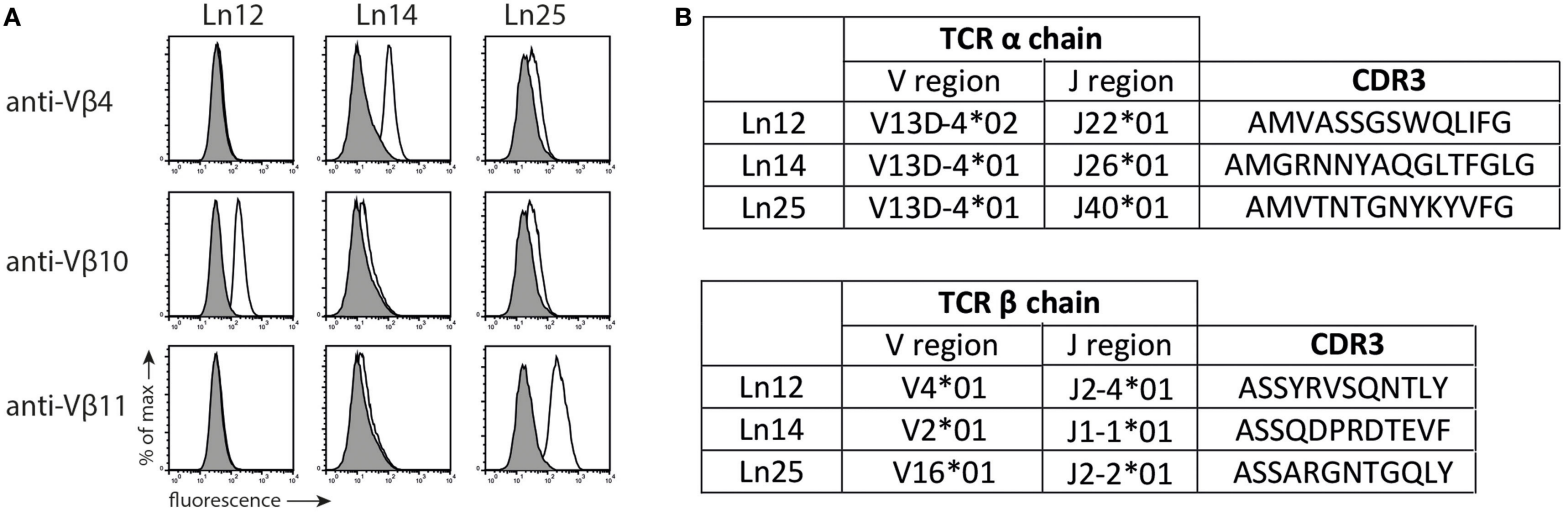
T cell receptor (TCR) usage of Qa-1^b^-restricted T cell clones. Three independent Qa-1^b^-restricted T cell clones were isolated (Ln12, Ln25, and Ln14). **(A)** TCRβ usage was determined by flow cytometry. Representative data from three independent experiments with similar results. **(B)** Amino acid sequence and CDR3 region of the TCRα and TCRβ chains, according to IMGT nomenclature. Complete amino acid sequences are displayed in Figure S1 in Supplementary Material.

Previously, we described the generation of Qa-1^b^-restricted T cell responses specific for target cells that lack the peptide transporter TAP (27). We investigated the diversity of three of these independently isolated CD8^+^ TCRαβ^+^ T cell clones (Ln12, Ln14, and Ln25) using a panel of TCR-Vβ-specific antibodies (Figure 1A). Each T cell clone expressed a different V-segment of the TCRβ chain: Vβ10 (ln12), Vβ4 (Ln14), and Vβ11 (Ln25), confirming their independent origin. Cloning and sequencing the TCRβ showed that the J- and D-segments as well as the CDR3 regions were also different (Figure 1B; Figure S1 in Supplementary Material). Interestingly, all three T cell clones expressed the same V-gene segment of the TCRα chains, namely TRAV13D-4 (Figure 1B). The J-segments and CDR3 regions of the TCRα chains, however, were different for each T cell. Thus, the TCR usage of these three Qa-1^b^-restricted T cell clones was generally diverse, but conserved in the Vα segment. Interestingly, limited TCR Vα diversity was recently also described for Qa-1^b^-restricted T lymphocytes specific for ERAAP-deficient target cells, although another Vα usage was reported for these CD8^+^ T cells (32).

### TAP-Deficiency Induces Presentation of Peptide Ligands in Splenocytes and Dendritic Cells

**Figure 2:**
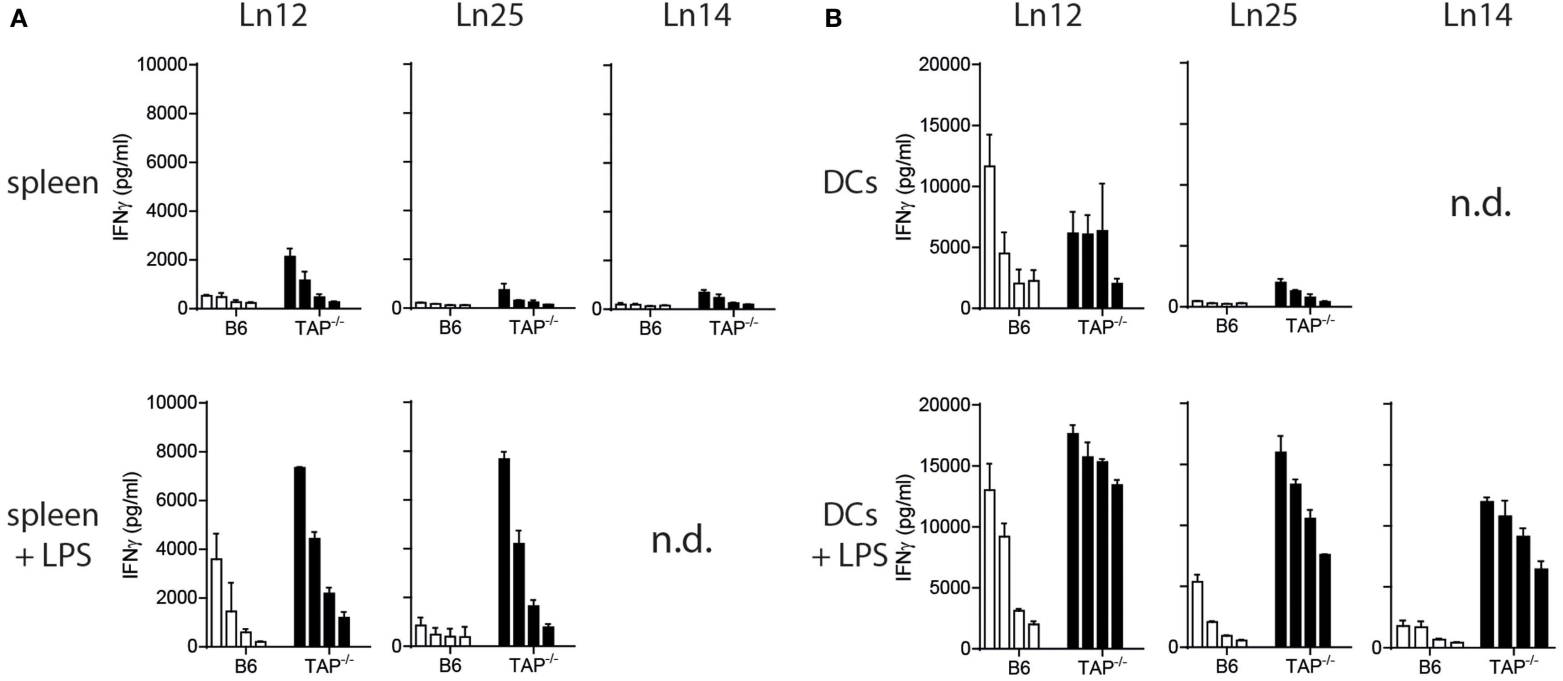
Splenocytes and dendritic cells from TAP1^−/−^ mice present non-mutated neoantigens. T cell clones were tested against titrated numbers of splenocytes **(A)** and dendritic cells **(B)** from wild-type B6 or TAP1^−/−^ mice. The TLR4 ligand LPS was added to target cells to activate them. A twofold titration of target cells was tested, starting with 20,000 cells (splenocytes) or 10,000 cells (DC) per well. IFNγ release by T cells was determined as a measure of reactivity. Data are shown as mean with SD of triplicates from one out of two similar experiments. n.d., not determined.

The absence of the peptide transporter TAP has an extensive impact on the surface levels of classical MHC class I molecules (33). We determined the consequence of TAP-deficiency for the nonclassical Qa-1^b^ and examined surface display on freshly isolated splenocytes and *in vitro* cultured bone-marrow derived dendritic cells (Figure S2 in Supplementary Material). Although Qa-1^b^ levels were indeed lower on TAP1^−/−^ cells compared to wild type, the difference was very small. Activation of the splenocytes and dendritic cells by the TLR-4 ligand LPS even further decreased this difference. Presentation of the dominant Qdm peptide is known to be dependent on the peptide transporter (3, 34), thus the Qa-1^b^ molecules on the surface of TAP1^−/−^ cells have to be filled with an alternative TAP-independent peptide repertoire of ‘self’ origin. We tested our three T cell clones for reactivity against these non-transformed target cells. Even though these CD8^+^ T cell clones had been generated against TAP-deficient tumor cells, they were able to recognize non-transformed splenocytes and dendritic cells of TAP1^−/−^ background (Figure 2). Short stimulation of TAP-deficient splenocytes with LPS resulted in strongly increased IFNγ release by the T cells (Figure 2A). Comparable results were obtained with LPS pretreated dendritic cells (Figure 2B), indicating that activation of antigen-presenting cells leads to increased reactivity by Qa-1^b^-restricted CD8^+^ T cells. This effect might partially be explained by higher Qa-1^b^ levels at the cell surface (Figure S2 in Supplementary Material). From these data, we concluded that all three independent T cell clones specifically recognized natural peptide ligands presented by non-transformed TAP-deficient target cells.

### Qa-1^b^-Presented Peptide Ligands Are Conserved across Species

**Figure 3:**
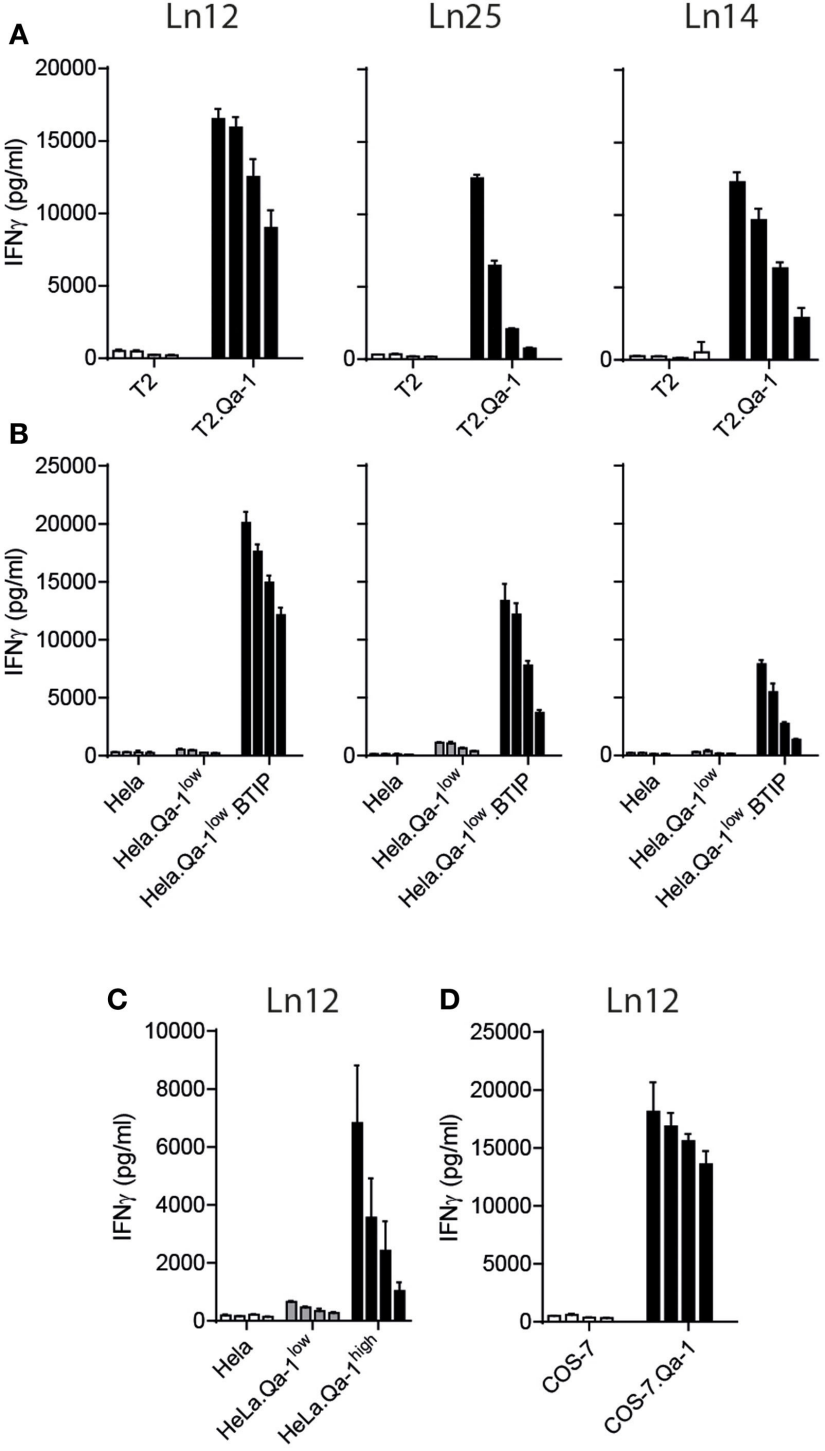
Qa-1^b^-restricted CD8^+^ T cell recognize conserved peptides from human and monkey. T cell clones were tested against human T2 cells and HeLa cells and monkey COS-7 cells. **(A)** Human TAP-negative T2 lymphoma cells were transduced with the gene for Qa-1^b^. **(B)** Human HeLa cells were transduced with the Qa-1^b^ gene and the viral TAP-inhibitor BTIP (=UL49.5). **(C)** TAP-proficient HeLa cells were selected for low or high surface expression of Qa-1^b^. **(D)** Monkey COS-7 cells were rendered Qa-1^b^ positive by gene transfer. Twofold titration of target cells are depicted, starting with 20,000 cells per well and T cell activation was measure *via* IFNγ production. Data are representative for at least two independent experiments with comparable results, shown as mean and SD of triplicates.

Next, we assessed the nature of the TAP-independent peptide ligands by transfection of the mouse Qa-1^b^ gene *H-2T23* into TAP-deficient human target cells (Figure 3). The mouse T cell clones were, therefore, tested against human T2 cells, which lost the chromosome locus coding for *TAP1* and *TAP2*, and human HeLa cells rendered TAP-deficient through a TAP-inhibition protein (BTIP) coded by the viral *UL49.5* gene (35). Interestingly, all three T cell clones showed vigorous reactivity to Qa-1^b^-positive TAP-deficient human cells (Figures 3A,B). Parental T2 and HeLa cells were not recognized, nor HeLa cells expressing only Qa-1^b^. Interestingly, the absence of the peptide transporter was not required when higher levels of Qa-1^b^ were introduced in HeLa cells (Figure 3C, Qa-1^high^). With five times higher surface Qa-1^b^ (Figure S2B in Supplementary Material) also the TAP-proficient HeLa cells were recognized by the T cell clone (Figure 3C). We hypothesize that saturating availability of Qa-1^b^ heavy chains consumed all Qdm peptides and thereby facilitate presentation of the alternative peptides. Finally, we found also T cell reactivity against COS-7 cells, which are kidney cells from an African green monkey (Figure 3D). Together, these data demonstrated the conserved nature of the T cell peptide-epitopes presented by Qa-1^b^, even though exact sequences were not identified.

### Qa-1^b^-Restricted TCR Have an Extended Interface with Peptide Residues

**Figure 4:**
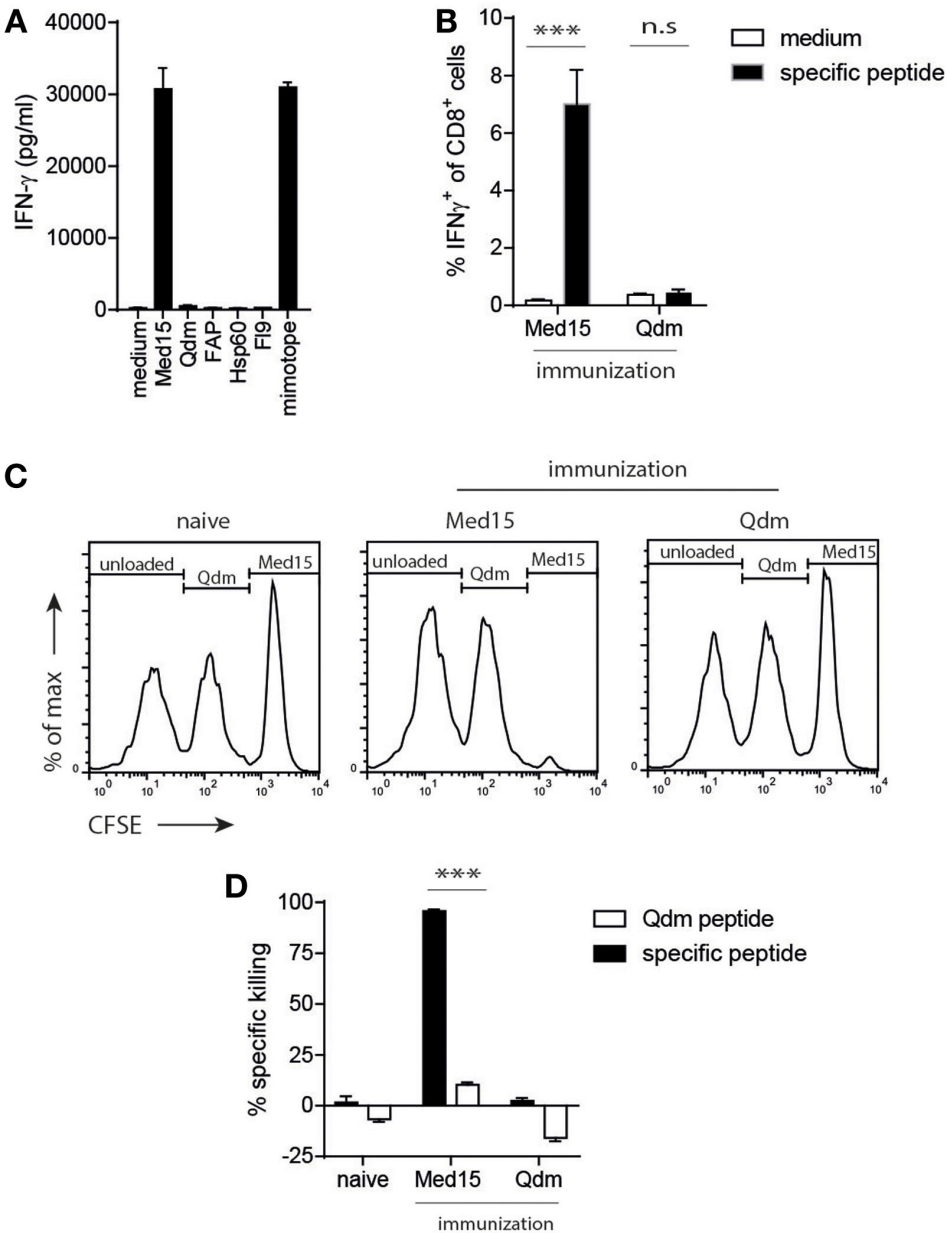
The Ln12 T cell peptide-epitope Med15 is immunogenic. The peptide derived from the Med15 cofactor for RNA polymerase II with sequence RLIIHFRDI is recognized by Ln12 T cells and immunogenic in C57/BL6 mice. **(A)** Ln12 T cell clone was tested against the Med15 peptide and other previously reported Qa-1^b^-binding peptides. **(B)** Med15 or Qdm peptides were used for immunization of C57/BL6 mice. Five days after the booster immunization, blood samples were taken and frequencies of CD8^+^ T cell responses were measured by intracellular IFNγ staining after brief *ex vivo* stimulation with immunized peptide or medium. Shown are means and SEM from eight mice. **(C,D)** Immunized C57/BL6 mice were challenged with a mix of three different splenocyte populations that were peptide-loaded and differentially labeled with CFSE. Splenocytes were injected at day 5 after booster immunization and harvested at day 7. Specific *in vivo* killing of these labeled target cells was calculated using the unloaded splenocyte subset as reference. Compiled data (*n* = 9) of three independent experiments with similar results are shown as mean with SEM. Student’s *t*-test was used for statistical analyses.

We aimed to identify the cognate peptide-epitopes of our T cell clones at the molecular level. None of the known Qa-1^b^-presented T cell epitopes were recognized by our T cells (Figure 4A). Thus, a synthetic bead-assisted peptide library with a complexity of 650,000 different 9-mer peptides was screened with the Ln12 T cell clone. This screen yielded an agonistic mimotope peptide with sequence ILIYHFRGV. To map the promiscuity of the TCR for Qa-1^b^-bound peptide ligands, each position of this mimotope sequence was exchanged for all other amino acids, and this collection of 171 synthetic peptides was tested in titrating concentrations for stimulation of the T cell clone Ln12 (Table S1 in Supplementary Material). Most amino acid replacements completely abolished T cell recognition, especially positions 2, 3, and 5 were very critical and nearly all alternative amino acids turned the mimotope into non-stimulating ligands. Positions 1 and 8 were indifferent for the TCR and nearly all other amino acids were allowed, or even improved, T cell reactivity. Position 2 and 9 have been described as dominant anchor positions for binding to the groove of Qa-1^b^ with preferred amino acids L and M (7, 36), suggesting that positions 3, 4, 5, 6, and 7 influenced the TCR interface. Thus, the Ln12 TCR is very peptide selective and does not merely engage Qa-1^b^ heavy chains when stabilized by any Qa-1^b^-binding peptide.

### The Conserved Med15-Derived Peptide Is a Ligand for Ln12 T Cells

The susceptible amino acids at each position that allowed activation of the Ln12 TCR, as depicted in Table 1, were used for a pattern homology search in the mouse proteome database in order to select naturally occurring agonistic peptides. The search with this “TCR motif” yielded 82 natural peptide sequences that were synthesized and tested in titrating concentrations for recognition by the Ln12 T cell clone (Table S2 in Supplementary Material). Out of these 82 naturally occurring peptides, only 6 stimulated the T cells at concentrations lower than 100 ng/ml and only one (RLIIHFRDI) was reactive as low as 100 pg/ml. This optimal peptide was derived from the Mediator complex subunit 15 (Med15), a cofactor involved in RNA polymerase II-dependent transcription. Interestingly, this peptide sequence was completely conserved in humans, underlining the finding that human cells were recognized upon Qa-1^b^ expression by Ln12 T cells (Figure 3). Of note, although the Ln25 and Ln14 T cell clones also recognized the human target cells, they did not react to the Med15 peptide, indicating that these two Qa-1^b^-restricted T cell clones recognize yet other peptide ligands.

**Table 1:** T cell receptor specificity.

aa position								
1	2	3	4	5	6	7	8	9
I	L	I	Y	H	F	R	G	V^a^
R	L	I	I	H	F	R	D	I^b^

A	L	I	F	H	C	I	A	F
C		V	I	I	F	K	C	I
D			L	Y	M	M	D	L
E			V		Q	P	E	M
F			Y		V	R	F	V
G						V	G	
H							H	
I							I	
K							L	
L							M	
M							N	
N							P	
P							Q	
Q							R	
R							S	
S							T	
T							V	
V							W	
W							Y	
Y								

*^a^All positions of the agonistic peptide ILIYHFRGV were replaced and tested with Ln12 T cells (Table S1 in Supplementary Material), resulting in the indicated susceptible aa*.

*^b^All natural peptides fulfilling this motif were tested with Ln12 (Table S2 in Supplementary Material), yielding the Med15 peptide (RLIIHFRDI)*.

### Strict Central Tolerance for T Cells to Qdm, but Not to the Med15 Ligand

Next, we determined if the identified Med15 peptide could also stimulate CD8^+^ T cells from the natural repertoire *via* vaccination. Up to 7% of CD8^+^ T cells responded to Med15 after peptide vaccination, whereas the Qdm peptide failed to induce a T cell response (Figure 4B). This vaccination-induced T cell immunity was cytotoxic, as CFSE-labeled and peptide-loaded splenocytes were efficiently and selectively killed *in vivo* (Figures 4C,D). As controls, naïve mice did not kill Med15-loaded targets and Med15-immunized mice did not kill Qdm-loaded targets, indicating that bona fide cytotoxic CD8^+^ T cells were elicited by peptide vaccination. The fact that wild-type mice are tolerant for Qdm-specific responses underlines its dominant presentation in homeostasis and strong clonal T cell deletion in the thymus. Together, these data imply that 1. the Ln12 TCR targets a specific peptide ligand and not merely stabilized Qa-1^b^ molecules; 2. the degree of degeneracy of this TCR is comparable to that of conventional T cells; and 3. each Qa-1^b^-restricted T cell clone targets a different agonistic peptide. These characteristics are largely reminiscent of conventional hypervariable CD8^+^ T cells, but not of invariant T cells like NKT and MAIT.

### Thymus Selection of Ln12 TCR is Qa-1^b^-Independent

**Figure 5:**
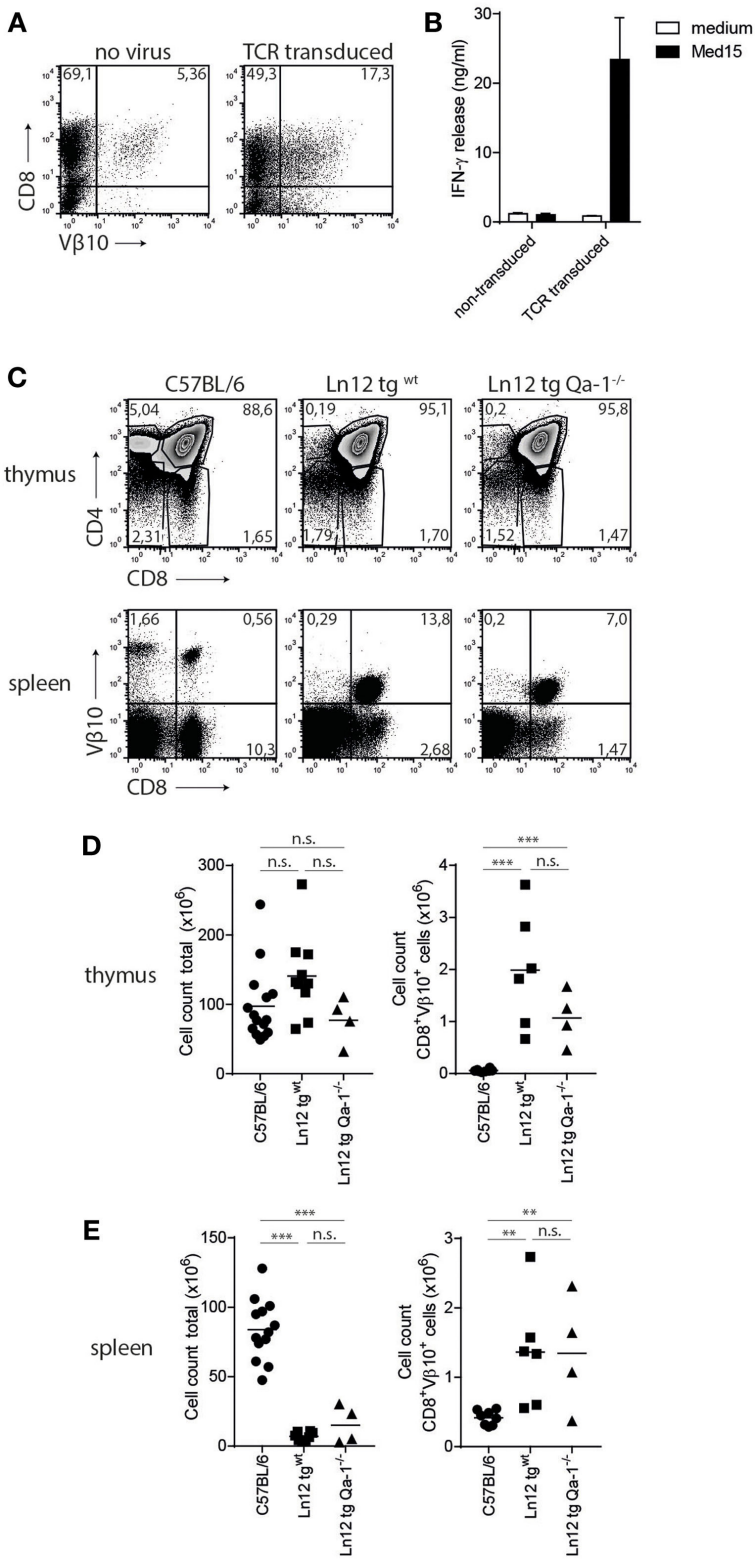
Selection of Ln12 T cell receptor (TCR)-transgenic cells is independent of Qa-1^b^. Generation and thymus selection analysis of a TCR-transgenic mouse based on the Ln12 TCR. **(A)** Splenocytes were retrovirally transduced with cloned alpha and beta (Vβ10) chains of the Ln12 T cell clone. **(B)** Transduced splenocytes were stimulated with Med15 peptide *in vitro* and IFNγ release by splenocytes was measured. Data representative of three independent experiments with comparable results and shown as mean with SD of triplicates. **(C)** Representative flow cytometry plots of thymus and spleen of C57/BL6, Ln12 tg ^WT^ and Ln12 tg Qa-1^−/−^ mice. Ln12 tg were both backcrossed to RAG1^−/−^. **(D,E)** Cellularity of thymus and spleen and absolute counts of Vβ10^+^ CD8^+^ T cells in thymus and spleen are depicted. One-way ANOVA was used for statistical analyses.

To study the thymus selection of Qa-1^b^-restricted Ln12 T cells, a TCR transgenic mouse was generated under control of the early CD2-based promoter. Transient expression of the cloned TCRαβ in splenocytes resulted in surface display and Med15-directed reactivity (Figures 5A,B), confirming correct cloning of the genes and demonstrating that the TCR confers the reactivity of Ln12 T cells. TCR transgenic mice (hereafter called Ln12 tg) were crossed on a Rag1^−/−^ background to prevent expression of endogenous TCRs and flow cytometry analysis was performed on the thymuses (Figures 5C,D). The frequency of immature CD4^+^CD8^+^ double positive thymocytes was increased in the Ln12 tg thymus, mostly due to absence of mature CD4 single positive T cells, demonstrating that the CD4 molecule does not support selection of Qa-1^b^-restricted T cells. Although the frequency of mature CD8 single positive thymocytes was not increased in the TCR tg (Figure 5C), the Vβ10^+^ CD8^+^ T cells were significantly increased (Figure 5D), suggesting inefficient positive selection. Total T cell numbers in the spleen were indeed low even tough thymus cellularity was comparable to RAG1^+/+^ wild-type mice (Figures 5C–E). Apparently, a low number of CD4^+^CD8^+^ double positive thymocytes developed into mature CD8 single positive T cells that populate the secondary lymph organs.

Importantly, no differences were observed in the thymus of Ln12 tg mice when backcrossed to Qa-1^b−/−^ mice (“Ln12 tg Qa-1^b−/−^”) (Figure 5C). Very similar percentages of immature CD4^+^CD8^+^ thymocytes and mature CD8^+^ T cells were detected (Figure 5D). In the spleen of these mice, clear and comparable populations of TCR-Vβ10^+^ CD8^+^ transgenic T cells were present in Qa-1^b−/−^ and Qa-1^b+/+^ mice (Figure 5E). Total cell counts of Ln12 tg cells in thymus and spleen of these two strains were also the same (Figures 5D,E), demonstrating that thymus selection of Ln12 tg T cells is independent of Qa-1^b^. We hypothesize that Ln12 tg T cells are selected on classical MHC class I molecules presenting a highly diverse peptide repertoire in the thymus and not on Qa-1^b^, which is strongly monomorphic with its Qdm peptide. Positively selected T cells then subsequently target Qa-1^b^ plus peptide in the periphery.

### Ln12 tg T Cells Largely Egress the Thymus As Naïve Cells and Display Effector Functions after Activation

**Figure 6:**
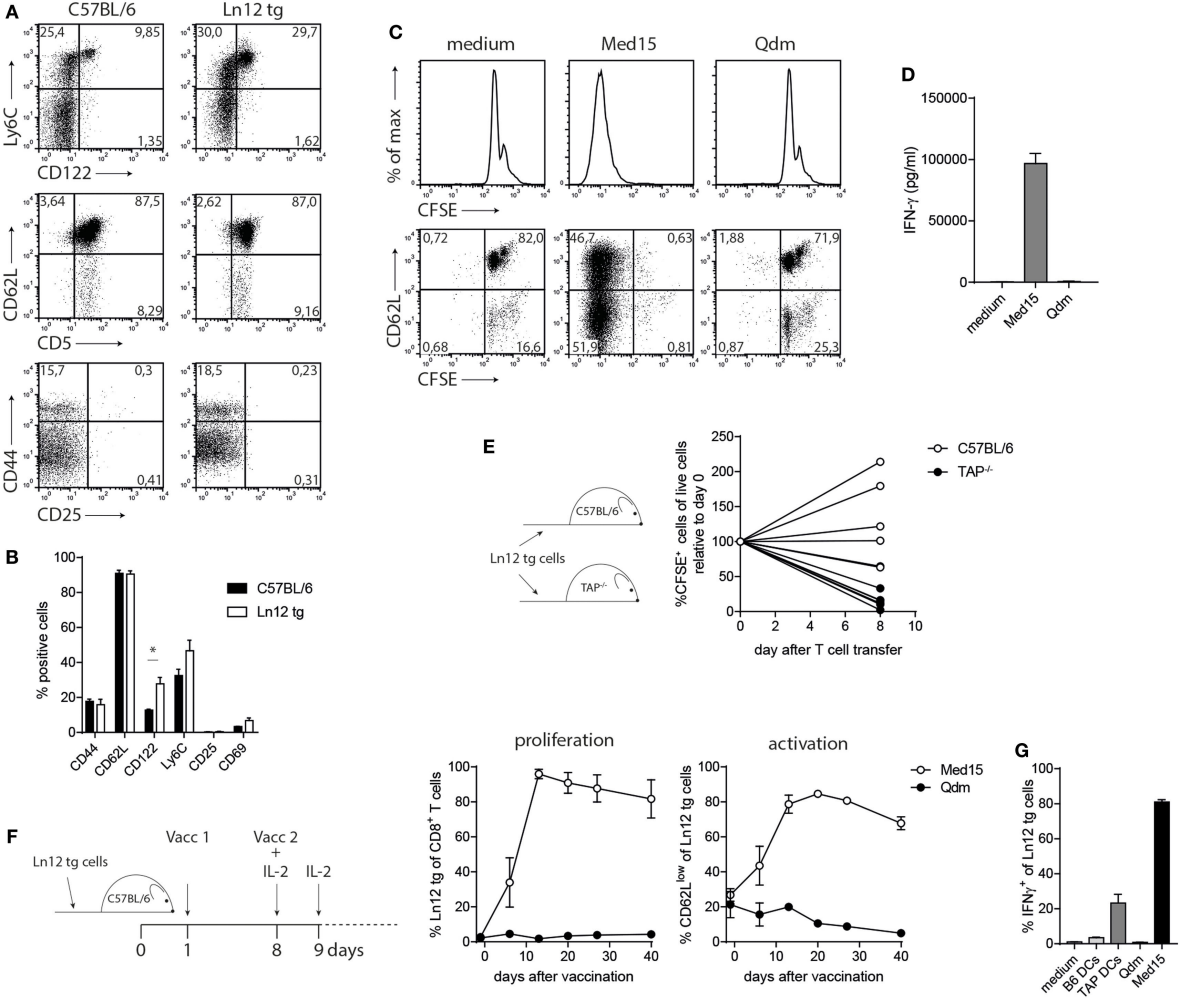
Normal behavior of peripheral T cell receptor (TCR)-transgenic Ln12 T cells. Mature peripheral T cells of the TCR-transgenic mouse were examined. **(A,B)** Expression of activation markers on CD8^+^ T cells in spleens of C57BL/6 or Ln12 tg mice was measured by flow cytometry. Combined data from at least eight animals are depicted, shown as mean with SEM of single markers. Student’s *t*-test was used for statistical analyses. **(C,D)** *In vitro* stimulation of purified CD8^+^ T cells from Ln12 tg mice with Med15 or Qdm peptide. Proliferation and IFNγ release were measured. Data are representative of at least two independent experiments. **(E)** Naïve CFSE-labeled CD8^+^ T cells from Ln12 tg mice were i.v. transferred into C57BL/6 or TAP1^−/−^. After 8 days, blood of recipient mice was analyzed for the frequency of Ln12 tg T cells. Data shown from one of the three experiments. Each line represents an individual mouse. **(F,G)** Ln12 tg CD8^+^ T cells were transferred to C57BL/6 mice and subsequently vaccinated with Med15 or Qdm peptide. Frequency and activation status was followed in blood of recipient mice with the use of a congenic marker. Data shown are mean with SEM of three mice and one of the two comparable experiments is shown. **(G)** Intracellular IFNγ detection in purified T cells was measured by flow cytometry upon brief *ex vivo* incubation with dendritic cells (DC) or peptides.

We examined the phenotype and function of the TCR transgenic Qa-1^b^-restricted T cells in more detail. Peripheral T cells in the Ln12 tg mice displayed normal frequencies of CD44, CD62L, CD25, and CD69, witnessing a naïve phenotype (Figures 6A,B). An increased population of CD122 and a small enhancement of Ly6C-positive cells indicated an antigen-experienced phenotype, but these small increases were minute compared to T cell subsets with “agonist selection,” like self-reactive regulatory T cells, NKT, and MAIT cells (37). In addition, Ln12 tg T cells did not exhibit an activated status, as the cells did not proliferate *ex vivo* in culture medium (Figures 6C,D). Addition of the Med15 peptide, but not control Qdm peptide induced activation of the cells, leading to proliferation and IFNγ production (Figures 6C,D). Activation of the T cells *in vivo* was demonstrated by transfer into naïve C57BL/6 or TAP1^−/−^ mice (Figure 6E). Eight days after T cell transfer, the majority of the cells were not detected anymore in TAP1^−/−^ mice, whereas no loss of T cells was observed in the C57BL/6 hosts. Disappearance in TAP1^−/−^ mice was most likely the result of cognate antigen encounter in the absence of co-stimulation.

Finally, we investigated the potential of Ln12 tg T cells to respond to peptide vaccination. TCR-transgenic cells were transferred in wild-type hosts, which were subsequently vaccinated twice with the Med15 peptide or Qdm peptide (Figure 6F). Transferred T cells underwent massive expansion, reaching frequencies of up to 90% of the total CD8^+^ T cell pool in recipient mice, accompanied by an activated CD62L^low^ phenotype (Figure 6F). In contrast, Qdm peptide vaccination did not activate the cells. *In vivo* activated Ln12 tg T cells also exhibited reactivity to TAP-deficient dendritic cells and not to their TAP-proficient counterparts, similar to the parental Ln12 T cell clone (Figures 6G and 2B).

Overall, our results demonstrate that Qa-1^b^-restricted CD8^+^ T cells display characteristics of both conventional hypervariable T cells as well as invariant T cells and can be classified as semi-invariant T cells, with only conserved Vα usage, positioned between these two known T cell subsets.

## Discussion

In this study, we describe characteristics of the Qa-1^b^-restricted CD8^+^ T cell subset that are reminiscent of T cell subtypes targeting other conserved MHC class Ib molecules, like CD1d, MR1, and H2-M3. For some features, however, the Qa-1^b^ subset strikingly differs from these invariant NKT cells and MAIT T cells and resembles conventional hypervariable CD8αβ^+^ TCRαβ^+^ T cells. We postulate that the Qa-1^b^ T cell subset is closest to conventional CD8^+^ T cells of all other alternative MHC-Ib recognizing populations.

In three independent T cell clones, a single Vα segment was used in conjunction with diverse CDR3 sequences and completely different Vβ TCR chains. This variance is much higher than that found in NKT and MAIT cells where completely invariant TCRα chains are used, including specific Jα segments, also in combination with a limited set of Vβs (38, 39). These invariant receptors target very conserved and monomorphic antigens in categories as glycolipids and vitamin B metabolites (39, 40). As a result, these T cell subsets regularly display self-reactivity, similar to FoxP3^+^ natural T_reg_ cells, yet, another dedicated T cell lineage with specialized function (37, 41). The Qa-1^b^ T cell population we describe here are clearly distinct from these T cell subsets, as both TCRα and TCRβ chains harbor much more diversity and, additionally, a diverse set of peptide epitopes are recognized (see Figure 1). Recently, a very restricted usage of only TCRα chains was observed in a TCRβ-transgenic mouse with specificity for a Qa-1^b^-presented peptide was reported (32). Though the Vα element in that study was not the same as we found, together, these findings indicate that the monomorphic Qa-1^b^ molecule, normally presenting the dominant Qdm peptide in homeostasis (4) imposes a strong limitation to the pool of functional TCRs that engage this MHC class Ib molecule during the selection process. Qdm-reactive T cells were indeed efficiently removed from the normal T cell repertoire, most likely due to central tolerance (see Figure 4). Importantly, we showed that thymus selection of our Qa-1^b^ T cell specificity was independent of the presence of Qa-1^b^ (see Figure 5), indicating that these TCRs engage other MHC class I molecules for positive selection in the thymus and then “cross-react” to peptide/Qa-1^b^ complexes in the periphery. Such cross-reactivity between classical and non-classical MHC molecules was reported before (42, 43) and underlines the similarities of their structures (7). Together, these results implicate limited availability of suitable TCRs and, therefore, low T cell precursor frequencies, to target each peptide-epitope presented by Qa-1^b^.

The mentioned T cell subsets targeting other MHC class Ib molecules, like CD1d, MR1, and H2-M3, have been described to leave the thymus with an activated phenotype, expressing high levels of CD44, CD69, CD122, and Ly6C (37, 41, 44). It was postulated that these T populations are equipped with this activated phenotype and organ-homing capacities in order to quickly respond upon infections much like innate lymphoid cells that lack a TCR. This activated phenotype of recent thymic emigrants is related to a so-called “agonist selection” in the thymus, where strong TCR triggers results in an already antigen-experienced phenotype. Indeed, FoxP3^+^ natural T_reg_ cells, NKT cells, and CD8αα intraepithelial lymphocytes are T cell subsets with agonist selection (37). Interestingly, these subsets have been reported to undergo a unique selection process, in that, hematopoietic cells in the thymus are critical for TCR triggering and positive selection, in contrast to conventional CD8^+^ T cells where thymus epithelial cells are key (37, 44, 45). Analysis of our TCR-transgenic mouse also revealed a higher proportion of activated thymic emigrants, regarding expression of CD122 and Ly6C. However, classical markers of “agonist selection,” like CD44 and CD25, were not enhanced, and the overall phenotype was much less convincing compared to real invariant T cell subsets and closer resembled the pattern of conventional T cells. Our findings corroborate those reported for Qa-1^b^-restricted T cells targeting ERAAP-deficient targets (26, 32).

The last characteristic that is shared by most MHC class Ib T cell subsets is the monomorphic nature of their antigens (39, 40). Hence, this feature is directly related to their invariant TCR chains. The alternative peptide repertoire, however, of Qa-1^b^ emerging at the surface of cells with defects in their antigen-processing machinery, like ERAAP or TAP deficiency, is extensively diverse. Peptidome analyses revealed more than 80 different sequences in Qa-1^b^ and more than 500 in HLA-E, when Qdm is withhold from binding (27, 28). These findings are underscored by a recent study in rhesus macaques vaccinated with a CMV vector encoding SIV antigens. An unexpected large fraction of vaccine-induced CD8^+^ T cells were directed to MHC-E presenting a variety of SIV peptides (13). Thus, the human HLA-E molecule might very well serve as a non-polymorphic antigen presentation platform, allowing display of a multitude of peptide antigens. Importantly, our results on scanning the TCR interface using a collection of 171 sequences based on the mimotope agonist revealed a critical and broad interaction with the Qa-1^b^ bound peptide (see Table 1; Tables S1 and S2 in Supplementary Material). Several amino acid residues of the peptide were critical for proper T cell activation. Of interest, the reported structure of a human TCR interacting with a Qdm-like peptide in HLA-E showed a regular diagonal docking of the receptor, very similar to that of conventional TCRs (24), suggesting that a broad interaction with several peptide side chains is definitively possible. Finally, our *in vivo* results with the TCR-transgenic T cells closely mimic a T cell differentiation program of conventional T cells: they largely emerge from the thymus as naïve lymphocytes, require priming for activation, and undergo a typical clonal expansion and subsequent contraction phase, resulting in memory cells (see Figure 6). Together, these results predict that exploitation of the universal HLA-E antigen presentation platform for immunotherapy of viral disease or cancer is feasible and will recruit an alternative T cell subset, which is quite conventional in its behavior.

## Ethics Statement

All animal experiments were controlled by the animal welfare committee (IvD) of the Leiden University Medical Center and approved by the national central committee of animal experiments (CCD) under the permit number AVD116002015271.

## Author Contributions

Conception and design of the work (ED and TH); acquisition of data (ED, MS, BQ, US, and CO); analysis and interpretation of the data (ED, SB, and TH); writing the paper (ED and TH).

## Conflict of Interest Statement

The authors declare that the research was conducted in the absence of any commercial or financial relationships that could be construed as a potential conflict of interest.
