# Supplementary material for: T Cells Engaging the Conserved MHC Class Ib Molecule Qa-1b with TAP-Independent Peptides Are Semi-Invariant Lymphocytes
Source: Front Immunol. 2018 Jan 25;9:60. doi: 10.3389/fimmu.2018.00060 (PMC5788890; doi:10.3389/fimmu.2018.00060)
Supplement: Supplementary file 1 [file data_sheet_1.PDF]

Legends Supplementary Figures

Supplementary Figure S1: Amino acid sequences of three T cell receptors of Qa-1^b-restricted $CD8^+$ T cell clones

TCRs were cloned and sequenced by NGS deep sequencing. The deduced amino acid order of the three independent T cell clones is depicted.

Supplementary Figure S2: Qa-1^b expression of target cells

Expression of Qa-1^b on mouse (A) and human (B) target cells was determined by flow cytometry. Grey histograms are PBA controls. Geometric means of the fluorescence is indicated in the left corner of the histogram plots. The means of PBS controls were subtracted from those of the Qa-1^b stained samples.

Supplementary Figure S1

Ln12

TCRα:
MKRLLCSLLGLLCTQVCWLKEQQVQQSPASLVLQEAENAELQCSFSIFTNQVQWFYQRPGGRLVSLLYNPSGTKQSGRLTSTT
VIKERRSSLHISSSQITDSGTYLCAMVASSGSWQLIFGSGTQLTVMPDIQNPEPAVYQLKDPRSQDSTLCLFTDFDSQINVPKTM
ESGTFITDKTVLDMKAMDSKSNGAIAWSNQTSFTCQDIFKETNATYPSSDVPCDATLTEKSFETDMNLNFQNLSVMGLRILLLK
VAGFNLLMTLRLWSS

TCRβ:
MGCRLLSCVAFCLLGIGPLETAVFQTPNYHVTQVGNEVSFNCKQTLGHDTMYWYKQDSKKLLKIMFSYNNKQLIVNETVPRRF
SPQSSDKAHLNLRIKSVEPEDSAVYLCASSYRVSQNTLYFGAGTRLSVLEDLRNVTPPKVSLFEPSKAEIANKQKATLVCLARGFFP
DHVELSWWVNGKEVHSGVSTDPQAYKESNYSYCLSSRLRVSATFWHNPRNHFRCQVQFHGLSEEDKWPEGSPKPVTQNISA
EAWGRADCGITSASYHQGVLSATILYEILLGKATLYAVLVSGLVLMAMVKKKNS

Ln25

TCRα:
MKRLLCSLLGLLCTQVCWLKEQQVQQSPASLVLQEGENAELQCSFSIFTNQVQWFYQRPGGRLVSLLYNPSGTKQSGRLTSTT
VIKERRSSLHISSSQITDSGTYLCAMVTNTGNYKYVFGAGTRLKVIAHIQNPEPAVYQLKDPRSQDSTLCLFTDFDSQINVPKTME
SGTFITDKTVLDMKAMDSKSNGAIAWSNQTSFTCQDIFKETNATYPSSDVPCDATLTEKSFETDMNLNFQNLSVMGLRILLLKV
AGFNLLMTLRLWSS

TCRβ:
MAPRLLFCLVLCFLRAEPTNAGVIQTPRHKVTGKGQEATLWCEPISGHSAVFWYRQTIVQGLEFLTYFRNQAPIDDSGMPKERF
SAQMPNQSHSTLKIQSTQPQDSAVYLCASSARGNTGQLYFGEGSKLTVLEDLRNVTPPKVSLFEPSKAEIANKQKATLVCLARGF
FPDHVELSWWVNGKEVHSGVSTDPQAYKESNYSYCLSSRLRVSATFWHNPRNHFRCQVQFHGLSEEDKWPEGSPKPVTQNI
SAEAWGRADCGITSASYHQGVLSATILYEILLGKATLYAVLVSGLVLMAMVKKKNS

Ln14

TCRα:
MKRLLCSLLGLLCTQVCWLKEQQVQQSPASLVLQEGENAELQCSFSIFTNQVQWFYQRPGGRLVSLLYNPSGTKQSGRLTSTT
VIKERRSSLHISSSQITDSGTYLCAMGRNNYAQGLTFGLGTRVSVFPYIQNPEPAVYQLKDPRSQDSTLCLFTDFDSQINVPKTM
ESGTFITDKTVLDMKAMDSKSNGAIAWSNQTSFTCQDIFKETNATYPSSDVPCDATLTEKSFETDMNLNFQNLSVMGLRILLLK
VAGFNLLMTLRLWSS

TCRβ:
MGSIFLSCLAVCLLVAGPVDPKIIQKPKYLVAVTGSEKILICEQYLGHNAMYWYRQSAKKPLEFMFSYSYQKLMDNQTASSRFQ
PQSSKKNHLDLQITALKPDDSATYFCASSQDPRDTEVFFGKGTRLTVVEDLRNVTPPKVSLFEPSKAEIANKQKATLVCLARGFFP
DHVELSWWVNGKEVHSGVSTDPQAYKESNYSYCLSSRLRVSATFWHNPRNHFRCQVQFHGLSEEDKWPEGSPKPVTQNISA
EAWGRADCGITSASYHQGVLSATILYEILLGKATLYAVLVSGLVLMAMVKKKNS

Supplementary figure S2

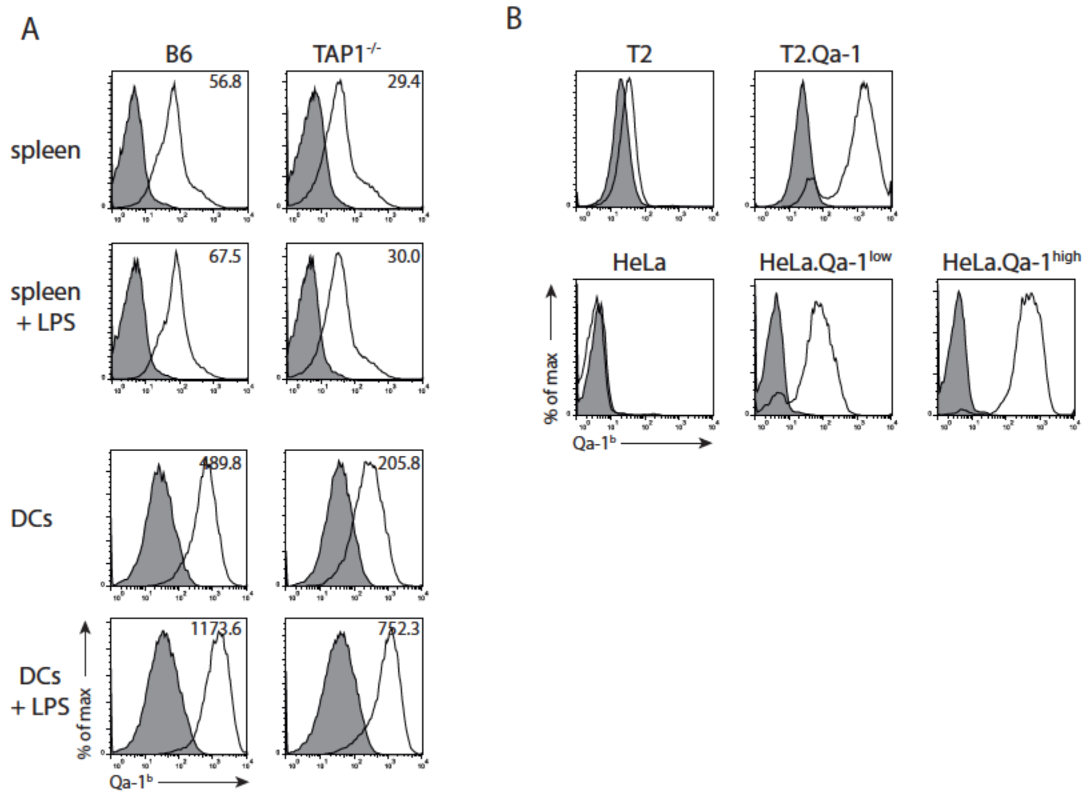

Suppl Table I. Position screen

	Amino acid position[a]								
	1	2	3	4	5	6	7	8	9
A	=[b]	0	0	<	0	0	<	=	<
C	=	0	0	<	0	=	<	=	<
D	=	0	0	<	0	0	<	=	0
E	=	0	0	0	0	0	<	>	<
F	=	0	0	=	0	-	<	=	=
G	=	0	0	0	0	0	<	-	<
H	=	0	0	<	-	0	0	=	0
I	-	0	-	=	=	<	>	=	=
K	>	0	0	0	<	0	=	<	0
L	=	-	0	=	<	<	<	=	=
M	=	0	0	<	0	=	=	>	=
N	=	0	0	<	0	<	<	>	<
P	=	0	0	0	0	<	>	>	0
Q	=	0	0	<	0	=	<	>	<
R	>	0	0	0	0	0	-	=	0
S	=	0	<	0	0	<	<	=	<
T	=	0	<	<	0	<	<	=	<
V	=	0	=	=	0	=	>	=	-
W	=	0	0	0	0	0	<	=	<
Y	=	0	0	-	=	<	<	>	0

[a] Each position in this matrix represents one single peptide comprising a substituted amino acid in the ILIYHFRGV mimotope sequence. These 171 peptides were tested with CTL in titrating concentrations.

[b] Results are depicted as capacity to stimulate Ln12 compared to mimotope: < worse, = equal, **>** better, 0 no stimulation, **-** original residue.

Suppl Table II. List of natural peptides encompassing the TCR motif[a]

peptide number	peptide sequence	Ln12 recognition (pM)[b]	peptide location	protein length	UniProtK / SwissProt number	Protein description
mimotope	ILIYHFRGV	100	--	--	--	selected mimotope sequence
1	RLIIHFRDI	100	61-69	749	Q7TQE1	Mediator complex subunit 15
2	SLIVHFRWL	1,000	2354-2362	3717	NP_001098012	Vomeronasal receptor Vmn2r93
3	LLILHFIGI	10,000	234-242	307	Q7M724	Taste receptor type 2 member 106
4	KLVIHCKDL	100,000	139-147	747	Q80TA6	Myotubularin-related protein 12
5	PLIIHVIHL	100,000	412-420	639	Q3USH9	Putative uncharacterized protein
6	DLVVHMKMV	1,000,000	318-326	645	Q8C0I1	Alkyldihydroxyacetonephosphate synthase, peroxisomal
7	GLVIIVPFL	1,000,000	204-212	315	Q8VEZ6	Olfactory receptor 397
8	KLIVHCKFL	1,000,000	965-973	2007	Q6P5D8	Structural maintenance of chromosomes flexible hinge domain-containing protein 1
9	QLILIMKSL	1,000,000	61-69	699	B2RQA7	Putative uncharacterized protein
10	ALVVICIVL	> 5,000,000	18-26	761	P97321	Seprase
11	ALVVYVVFF	> 5,000,000	338-346	484	O70451	Monocarboxylate transporter 2
12	CLIFIFPAI	> 5,000,000	443-451	505	Q9DCP2	Sodium-coupled neutral amino acid transporter 3
13	CLVLIVKLL	> 5,000,000	340-348	601	Q80SU6	Sodium-dependent phosphate transport protein 2C
14	DLSVIMILT	> 5,000,000	4-12	765	Q8C145	Zinc transporter ZIP6
15	DLVLIVVAV	> 5,000,000	590-598	659	Q8BH79	Anoctamin-10
16	DLVYYVRVT	> 5,000,000	953-961	2583	NP_001116075	Fc fragment of IgG binding protein
17	ELIIYCKDF	> 5,000,000	133-141	771	Q7TPM9	Myotubularin-related protein 10
18	ELVVYCRPV	> 5,000,000	955-963	1302	Q62077	Phospholipase C-gamma-1
19	FLIFHMVVL	> 5,000,000	344-352	558	Q8C4W1	Putative uncharacterized protein
20	FLIIYMISV	> 5,000,000	31-39	315	Q8VGS9	Olfactory receptor MOR186-1
21	FLIIYVVTV	> 5,000,000	32-40	312	Q8VGL1	Olfactory receptor MOR103-5
22	FLSFYMVTV	> 5,000,000	31-39	309	Q7TRE9	Olfactory receptor Olfr872
23	FLSIYFVTI	> 5,000,000	31-39	313	Q7TRG4	Olfactory receptor Olfr843
24	FLSLYMMAL	> 5,000,000	33-41	312	Q7TRT1	Olfactory receptor Olfr539
25	FLSLYVMAL	> 5,000,000	31-39	310	Q7TRT8	Olfactory receptor Olfr60
26	FLSVYFIIT	> 5,000,000	18-26	139	Q9CX13	Protein cornichon homolog 4
27	FLTLYMMAL	> 5,000,000	32-40	311	Q7TRT2	Olfactory receptor Olfr53
28	FLTLYVVTV	> 5,000,000	38-46	323	Q8VGU3	Olfactory receptor 1413
29	FLVIYFMTI	> 5,000,000	31-39	309	Q8VFB9	Olfactory receptor 183
30	FLVIYVVTM	> 5,000,000	29-37	305	Q8VGM8	Olfactory receptor MOR231-3
31	FLVLYMVTV	> 5,000,000	38-46	321	Q8VET3	Olfactory receptor 1412
32	FLVLYVVTI	> 5,000,000	38-46	322	Q8VFC5	Olfactory receptor MOR208-2
33	FLVLYVVTV	> 5,000,000	38-46	333	Q60894	Olfactory receptor 12
34	FLVMYVIAT	> 5,000,000	31-39	310	Q7TRC7	Olfactory receptor Olfr907
35	FLVVYMVTL	> 5,000,000	29-37	310	Q7TR85	Olfactory receptor Olfr1036
36	FLVVYVVTV	> 5,000,000	30-38	336	Q7TR87	Olfactory receptor Olfr1031
37	GLSLIFIAV	> 5,000,000	56-64	450	Q9D3A9	Protein tweety homolog 1
38	GLVFYVPLI	> 5,000,000	434-442	478	Q91WN3	Solute carrier family 7 member 13
39	GLVVIFMPL	> 5,000,000	281-289	304	XP_001472553	Hypothetical protein
40	HLTVIFKGM	> 5,000,000	21-29	241	Q8BI77	Putative uncharacterized protein
41	ILIFYMMTL	> 5,000,000	32-40	312	Q8VFC2	Olfactory receptor MOR256-18
42	ILIFYVPGM	> 5,000,000	251-259	316	Q7TRN7	Olfactory receptor Olfr685
43	ILIIIVPGV	> 5,000,000	202-210	490	Q9WTR6	Cystine/glutamate transporter
44	ILIVIFPIL	> 5,000,000	61-69	223	Q61735	Leukocyte surface antigen CD47
45	ILIVIFVLL	> 5,000,000	717-725	1115	P13595	Neural cell adhesion molecule 1
46	KLSVYMRRT	> 5,000,000	45-53	134	EDL32259	mCG140070
47	KLVFHVIII	> 5,000,000	539-547	560	Q8BGD6	Putative sodium-coupled neutral amino acid transporter 9
48	LLIIIFIVT	> 5,000,000	18-26	131	Q7TPG7	Protein FAM19A2
49	LLILYVVVV	> 5,000,000	21-29	793	Q9DC23	DnaJ homolog subfamily C member 10
50	LLIYIVKIV	> 5,000,000	30-38	307	Q8VG60	Olfactory receptor MOR231-10
51	LLVFICVAM	> 5,000,000	323-331	433	P59053	Potassium voltage-gated channel subfamily G member 3
52	LLVFIVILM	> 5,000,000	546-554	788	Q80TG9	Leucine-rich repeat and fibronectin type-III domain-containing protein 2
53	LLVIYFPPL	> 5,000,000	855-863	918	Q80XR2	Calcium-transporting ATPase type 2C member 1
54	LLVLHVKYL	> 5,000,000	68-76	326	Q8BUB3	Putative uncharacterized protein
55	LLVLYFRNF	> 5,000,000	36-44	709	Q9JIP7	Solute carrier family 15 member 1
56	LLVVIQIQT	> 5,000,000	12-20	74	Q64389	CAMPATH-1 antigen
57	MLIIIVICM	> 5,000,000	47-55	324	Q8VG22	Olfactory receptor MOR41-1
58	MLSFICVEL	> 5,000,000	267-275	1733	Q4H4D7	ATP-binding cassette (ABC) transporter ABCA14
59	NLILYQVDV	> 5,000,000	698-706	2874	Q8BW94	Dynein heavy chain 3, axonemal
60	NLVFYVVGL	> 5,000,000	79-87	349	Q640P4	Glycosyltransferase 8 domain-containing protein 2
61	PLIYYVPDF	> 5,000,000	21-29	238	Q8K2U2	Alkylated DNA repair protein alkB homolog 6
62	PLVLYVVPI	> 5,000,000	109-117	338	Q3KNA1	Mas-related G-protein coupled receptor member B2
63	QLVIYQVGL	> 5,000,000	11-19	519	Q80Y28	Abcd4 protein
64	RLTLIFVLI	> 5,000,000	9-17	167	Q3TDT7	Putative uncharacterized protein
65	SLIFYCIIV	> 5,000,000	6-14	795	Q9EPQ1	Toll-like receptor 1
66	SLVVIFMLV	> 5,000,000	697-705	975	NP_001074703	Phosphatidylinositol glycan anchor biosynthesis, class G
67	TLILHQVEL	> 5,000,000	14-22	241	Q8CI01	Proz protein
68	TLSMICPVL	> 5,000,000	521-529	550	XP_991169	Similar to Muc1
69	TLSVHCPLF	> 5,000,000	106-114	114	Q9D974	Putative uncharacterized protein
70	TLVYHVVGV	> 5,000,000	165-173	1217	Q921M3	Splicing factor 3B subunit 3
71	VLIFYCPTV	> 5,000,000	80-88	398	Q8C025	Cholinephosphotransferase 1
72	VLIFYVPII	> 5,000,000	263-271	328	Q6W055	Olfactory receptor Olfr576
73	VLIFYVPLI	> 5,000,000	250-258	318	Q8VGZ3	Olfactory receptor 572
74	VLIFYVPMV	> 5,000,000	129-137	193	Q8VEQ6	Olfactory receptor MOR13-5
75	VLIFYVPVI	> 5,000,000	253-261	317	Q8VH13	Olfactory receptor MOR8-2
76	VLILYVPMI	> 5,000,000	250-258	319	Q8VGZ8	Olfactory receptor MOR17-2
77	VLIYYVPMI	> 5,000,000	250-258	316	Q8VGY8	Olfactory receptor MOR12-1
78	VLVFYVPLI	> 5,000,000	259-267	322	Q8VF09	Olfactory receptor MOR18-3
79	VLVLHCRAL	> 5,000,000	1895-1903	2430	Q3UHA3	Spatacsin
80	VLVLYVPMV	> 5,000,000	256-264	321	Q8VH20	Olfactory receptor MOR5-1
81	VLVYYVPMI	> 5,000,000	248-256	312	Q8VF02	Olfactory receptor MOR12-5
82	WLSVYVVQL	> 5,000,000	64-72	418	XP_977708	Hypothetical protein

[a] The IPI mouse protein database was searched for 9-mer peptides fulfilling the TCR motif from Table I using pattern search. This yielded the 82 peptides listed in this table. All peptides were synthesized and tested for recognition by the Qa-1-restricted Ln12 T cell clone.

[b] Peptides were titrated starting at 5 uM, loaded on splenocytes and IFNy production by the Ln12 T cells was measured after 18 hours. Values indicate the peptide concentration at half maximal T cell stimulation.
